# Safety and Efficacy of PrabotulinumtoxinA (Nabota^®^) Injection for Cervical and Shoulder Girdle Myofascial Pain Syndrome: A Pilot Study

**DOI:** 10.3390/toxins10090355

**Published:** 2018-09-03

**Authors:** Da-ye Kim, Jae Min Kim

**Affiliations:** Department of Rehabilitation Medicine, Incheon St. Mary’s Hospital, College of Medicine, The Catholic University of Korea, Incheon 21431, Korea; 550059@cmcism.ac.kr

**Keywords:** botulinum toxin, injection, myofascial pain syndrome, cervical, shoulder

## Abstract

Myofascial pain syndrome is a common painful condition encountered in the general population. Previous studies evaluating the efficacy of botulinum toxin for the treatment of myofascial pain syndrome are limited, with variable results. This prospective study investigated the efficacy and safety of direct injection of Prabotulinumtoxin A (Nabota^®^) into painful muscle groups for cervical and shoulder girdle myofascial pain. Twelve patients with chronic myofascial pain syndrome of the neck and shoulder underwent an injection of Prabotulinumtoxin A. Painful muscles containing trigger points were injected in the mid-belly. Pain scores and quality of life measurements were assessed at baseline, as well as 6 weeks and 12 weeks post-injection. Safety and tolerability were also assessed. This trial is registered under clinical research information service (CRIS) number KCT0001634. Patients injected with Prabotulinumtoxin A showed a significant improvement in pain at 12 weeks (*p* < 0.001). At 6 weeks, the pain had not significantly improved compared with baseline (*p* = 0.063). However, at that time, 41.7% of patients were characterized as Prabotulinumtoxin A responders, with a 30% reduction in pain rating score compared to baseline. In the Neck Disability Index scores, the patients demonstrated significant improvement at both 6 weeks and 12 weeks. No serious adverse effects occurred during the study. Prabotulinumtoxin A injection into chronically painful muscles associated with cervical and shoulder girdle myofascial pain syndrome resulted in an improvement in pain scores and quality of life lasting at least 12 weeks. Additionally, the injections were well tolerated. As these are preliminary findings in a pilot study, future studies should carefully consider using randomized, controlled, prospective trials.

## 1. Introduction

Myofascial pain syndrome is a chronic pain syndrome caused by myofascial trigger points, most commonly those of the neck and shoulder. Treatments for myofascial pain syndrome include pharmacotherapy, physical therapy, therapeutic exercise, and trigger point injections [1].

Trigger point injections are treatments that directly target myofascial trigger points using local anesthetics. They are typically performed several times per week. However, botulinum toxin can be administered in a single injection that lasts for many months [2,3,4].

Zhou et al. [5] described the potential effects of botulinum toxin in myofascial pain syndrome, and Nicol et al. [4] reported that botulinum toxin improved pain and quality of life in patients with cervical and shoulder girdle myofascial pain syndrome.

Nabota^®^ is a botulinum toxin type A protein of high purity and quality developed by Daewoong Pharmaceutical in Korea, from the natural strain Clostridium botulinum (type A). Clinical trials have been conducted to demonstrate its effects on skin wrinkles and upper limb spasticity in stroke patients [6,7].

The purpose of the present study was to confirm the efficacy and safety of Prabotulinumtoxin A (Nabota^®^) injection in patients with cervical and shoulder girdle myofascial pain syndrome.

## 2. Results

In total, 12 patients with cervical and shoulder girdle myofascial pain syndrome were enrolled in this study. The demographic information and pain rating scales are presented in Table 1. The baseline characteristics of the patients according to the clinical variables are presented in Table 2.

**Figure 1:**
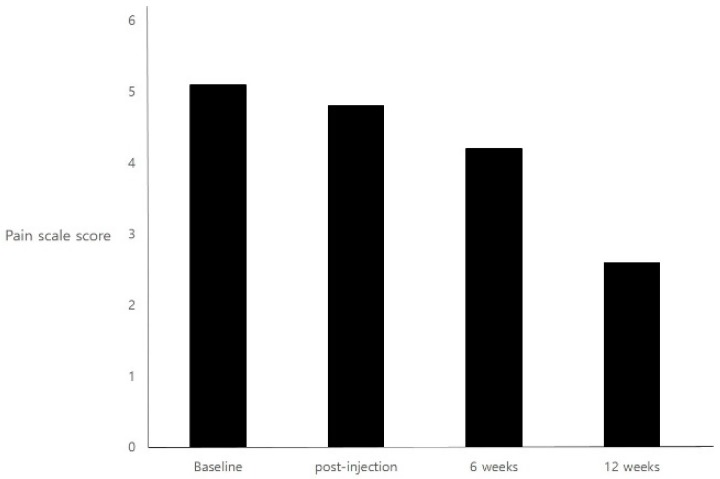
Mean score on the pain-rating scale at each assessment time point.

There was a statistically significant difference in pain scores after 12 weeks compared to baseline (*p* < 0.0001). However, there was no statistically significant difference either post-injection (*p* = 0.88) or after 6 weeks compared (*p* = 0.06) to baseline. The pain rating scale values were 5.1 ± 0.3, 4.8 ± 0.4, 4.2 ± 1.2 and 2.6 ± 1.3 at baseline, post-injection, 6 weeks, and 12 weeks, respectively (Figure 1). The proportion of patients whose pain scores improved by 30% or more was 8.33% at post-injection, 41.7% at 6 weeks, and 75% at 12 weeks.

**Figure 2:**
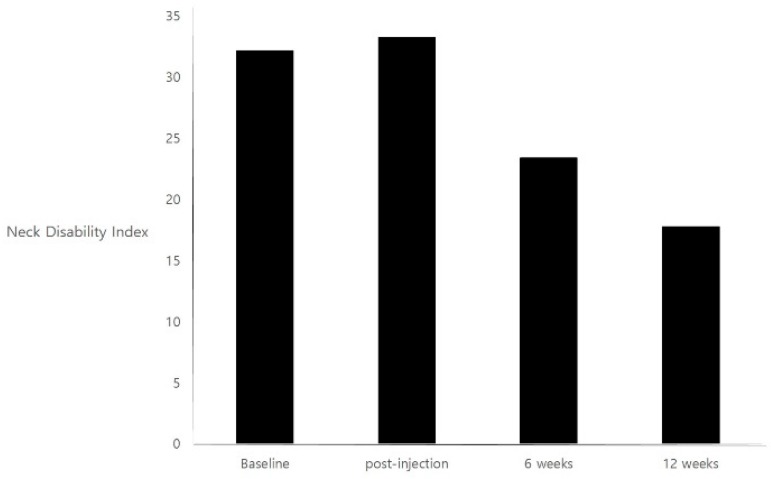
Mean score on the Neck Disability Index measuring quality of life at each assessment time point.

There was a statistically significant improvement in disability using the Neck Disability Index after 6 weeks (*p* = 0.01) and 12 weeks (*p* = 0.001) compared to baseline. However, there was no statistically significant difference post-injection (*p* = 0.37) compared to baseline. The Neck Disability Index values were 32.1 ± 11.9, 33.3 ± 32.3, 23.4 ± 14.7, and 17.8 ± 13.6 at baseline, post-injection, 6 weeks, and 12 weeks, respectively (Figure 2).

The average dose given to patients was 171.9 ± 25.1 units. A safety evaluation, including adverse events and adverse drug reactions, laboratory tests, vital signs, and physical and neurological examinations, was conducted for all patients who received the drug in this clinical trial. There were no adverse events reported regarding changes in vital signs and physical examination findings.

## 3. Discussion

This study evaluated the efficacy and safety of Prabotulinumtoxin A (Nabota^®^) in patients with cervical and shoulder girdle myofascial pain syndrome. A previous study [4] showed that botulinum toxin A injection improved pain scores and quality of life in patients with myofascial pain syndrome. We found that Nabota^®^ injection in patients with cervical and shoulder girdle myofascial pain syndrome significant decreased both pain scale and Neck Disability Index scores. There were no adverse events related to vital signs, physical and neurological examinations, and laboratory tests.

Our study results are similar to previous studies [2,3,4] of Botulinum toxin A injection for myofascial pain syndrome in relation to improvements in chronic pain. The pain rating score was significantly decreased from 5.1 to 2.6 at 12 weeks after the Nabota^®^ injection. A previous systemic review [8] documented that pain was reduced by Botulinum toxin A at 2 to 6 months after injection but not at 4 to 6 weeks. Our results showed that, in 75% of patients, the pain score had significantly improved at 12 weeks, and 41.7% of the patients had decreased pain scores at 6 weeks.

In a study by Nicol et al. [4], 50% of patients responded to injections of Botulinum toxin A in the first phase of the study. In our study, 41.7% of patients’ pain scores improved by at least 30% at 6 weeks. This shows that the efficacy of Nabota^®^ in myofascial pain syndrome is similar to that of Botulinum toxin A injection.

The results of the present study are consistent with those of earlier studies [3,4] that reported significant improvements in quality of life in patients with myofascial pain syndrome. We also found that Neck Disability Index scores, which were used to compare the degree of neck/shoulder function improvement, were statistically significantly improved after 6 and 12 weeks. The mean score was 32.1 before drug administration, 23.4 at 6 weeks after Nabota^®^ treatment, and 17.8 after 12 weeks.

Possible adverse effects related to botulinum toxin injection for myofascial pain syndrome include regional weakness, limb numbness, limb coldness, sore throat, nausea, flu-like symptoms, sore muscles, decreased systolic blood pressure, and arthralgia [2,3,4,9]. A previous study [7] reported adverse events of Nabota^®^ related to the musculoskeletal system, such as muscular weakness, muscle atrophy, and pain in the extremities. In the present study, there were no adverse reactions documented from the laboratory tests, vital signs, and physical and neurological examinations.

Prabotulinumtoxin A (Nabota^®^), a new botulinum toxin A originating from wild-type Clostridium botulinum A, presents higher purity of botulinum toxin A, which is confirmed by size exclusion high-performance liquid chromatography analysis [7]. This trial is the first study for safety and efficacy of Prabotulinumtoxin A (Nabota^®^) injection into painful muscle groups for myofascial pain.

The trigger point is defined as a palpable and hyperirritable nodule located in a taut band of muscle. Botulinum toxin A is effective in focal muscle hyperactivity [10]. In fact, electromyography activity at the trigger point demonstrates focal muscle hyperactive that play a significant role in the pain mechanisms [11]. Botulinum toxin A provides long lasting pain relief by causing prolonged muscle relaxation through inhibition of acetylcholine release. Botulinum toxin also inhibits the release of inflammatory mediators and peripheral neurotransmitters from sensory nerves [12]. These compounds are thought to be involved in peripheral and central sensitization in chronic myofascial pain. In the present study, the mean pain duration of the subjects was 20.3 months. Patients with chronic myofascial pain were effectively treated by botulinum toxin injection.

## 4. Study Limitations

The main limitation of this study was the relatively small number of patients. Although other preliminary studies have used fewer subjects and no controls, it is difficult to reach definite conclusions using this data set.

## 5. Conclusions

In conclusion, Nabota^®^ injection improved pain and quality of life after 12 weeks in patients with chronic cervical and shoulder girdle myofascial pain syndrome. This study confirmed that Nabota^®^ injection was a safe procedure for all of the patients. As this study is a preliminary study, we expect that further inquiries should carefully consider using randomized, controlled, prospective trials.

## 6. Material and Methods

### Subjects

This study was conducted with the approval by the Catholic University of Korea, Incheon St. Mary’s Hospital Institutional Research Review Board (IRB No. OC14MISI0102). This clinical trial was registered with the Clinical Research Information Service (CRIS, https://cris.nih.go.kr/cris/en/) under number (KCT0001634), approved on 23 September 2015. Twelve patients were enrolled in this prospective study, all of whom agreed in writing to voluntarily participate in the clinical trial and met the screening criteria. The inclusion criteria were as follows: (1) Male or female subjects no younger than 20 years; (2) myofascial pain of the neck and shoulders of at least 3-months duration; (3) failed standard therapy (i.e., physical modality, exercise, medication, and trigger point injections other than botulinum toxin A); (4) pain scale score of 5 or higher, and (5) voluntary informed consent signed by the patient after the objectives and methods of the clinical study had been explained. The exclusion criteria were as follows: (1) Patients with a history of neuromuscular disease; (2) patients receiving botulinum toxin A therapy within the past 4 months; (3) patients with a known allergy or sensitivity to any component of the test medication; (4) subjects who were pregnant or breastfeeding; (5) patients with significant atrophy of the muscles in the target injection area; (6) patients with muscle weakness in the target area (i.e., a Medical Research Council score < 5); (7) subjects deemed inappropriate for entry into this study in the judgement of the investigator; (8) patients taking muscle-relaxant drugs within 4 weeks prior to the screening visit; (9) patients taking aspirin, nonsteroidal anti-inflammatory drugs or antiplatelet drugs; (10) patients with another active illness; (11) patients with a moderate degree of cardiac disease or pulmonary disease; (12) patients with a herniated cervical nucleus pulposus, a straight cervical spine or a cervical sprain, and (13) patients who had participated in an investigational drug study or bioequivalence study within 3 months prior to the screening visit.

For the injections, one vial of Nabota^®^ (100 Units) was diluted with 4 mL of saline. The painful muscle containing the trigger point was injected with up to 50 Units of Nabota^®,^ and a maximum of 200 Units was allowed for each patient. The target muscle and dose for each injection were selected by a single skilled physician. The injection sites and doses for each muscle are shown in Table 3.

All patients received follow-up visits at 6 and 12 weeks after the injection. At each visit, the safety and efficacy of the drug were evaluated through a physical examination as well as a numerical rating scale for pain and a Neck Disability Index. The primary outcome measure was the percentage of patients whose pain scores (numerical rating scale) improved by 30% or more at 6 and 12 weeks after the injection compared to baseline. Disability as a secondary outcome was quantified using the Neck Disability Index.

Statistical analyses were performed using SAS^®^ software, version 9.3 (Cary, NC, USA). Paired *T*-test or Wilcoxon signed-rank test were used to evaluate differences in the pain scale and Neck Disability Index. A *p*-value < 0.05 was considered statistically significant.

## Figures and Tables

**Table 1:** Patient demographic information and pain rating scales.

Patient	Age (years)	Gender	Height (cm)	Weight (kg)	Duration of Pain (Months)	Pain Rating Scale			
						Baseline	Post-Injection	6 Weeks	12 Weeks
1	26	Female	158	41	10	5	5	5	5
2	42	Female	165	63	10	5	4	3	1
3	33	Female	170	68	24	5	5	3	2
4	36	Female	161	56	60	5	5	5	2
5	35	Female	160	56	10	6	5	4	3
6	39	Female	167	61	24	5	5	5	3
7	58	Female	154	51	24	5	5	5	4
8	44	Female	159	58	30	5	8	2	1
9	36	Female	162	53	12	5	5	5	3
10	22	Female	163	63	12	5	5	5	2
11	23	Female	162	58	12	5	5	2	1
12	24	Female	160	57	15	5	3	5	4

**Table 2:** Baseline characteristics of the patients.

Variable	Value (Mean ± Standard Deviation)
Age	34.8 ± 10.4
Female Gender	12 ^a^
Height (cm)	161.8 ± 4.2
Weight (Kg)	57.1 ± 6.9
Duration of Pain (Months)	20.3 ± 14.3

^a^ presented as number; The mean age was 34.8 ± 10.4 years, all of the patients were female, and none had previous injections of botulinum toxin.

**Table 3:** Injection sites and dose for each muscle.

Patient	Anterior Musculature (Units)				Posterior Musculature (Units)				
	Anterior Scalenes	Middle Scalenes	Pectoralis Major	Sternocleidomastoid	Levator Scapulae	Trapezius (Anterior Border)	Trapezius (Main Body)	Splenius Capitis	Semispinalis Capitis
1	6.25	6.25				6.25	25	125	6.25
2	6.25	6.25		6.25		25	25	6.25	6.25
3	6.25	6.25				25	25	6.25	6.25
4	6.25	6.25	12.5			25	25	6.25	6.25
5	6.25	6.25		6.25	6.25	25	25	6.25	6.25
6	6.25	6.25				25	25	6.25	6.25
7	6.25	6.25	12.5			25	25	6.25	6.25
8	6.25	6.25	12.5		12.5	25	25	6.25	6.25
9					12.5	25	50	6.25	6.25
10	6.25	6.25				25	50	6.25	6.25
11	6.25	6.25				25	50	6.25	6.25
12	6.25	6.25				25	25	6.25	6.25
